# Curvature sensing amphipathic helix in the C-terminus of RTNLB13 is conserved in all endoplasmic reticulum shaping reticulons in *Arabidopsis thaliana*

**DOI:** 10.1038/s41598-021-85866-3

**Published:** 2021-03-18

**Authors:** Rhiannon L. Brooks, Chandni S. Mistry, Ann M. Dixon

**Affiliations:** 1 MAS Centre for Doctoral Training, University of Warwick, Coventry, CV4 7AL UK; 2 Department of Chemistry, University of Warwick, Coventry, CV4 7AL UK

**Keywords:** Membrane biophysics, Membrane structure and assembly, Molecular biophysics, Bioanalytical chemistry, Circular dichroism, Solution-state NMR

## Abstract

The reticulon family of integral membrane proteins are conserved across all eukaryotes and typically localize to the endoplasmic reticulum (ER), where they are involved in generating highly-curved tubules. We recently demonstrated that Reticulon-like protein B13 (RTNLB13) from *Arabidopsis thaliana* contains a curvature-responsive amphipathic helix (APH) important for the proteins’ ability to induce curvature in the ER membrane, but incapable of generating curvature by itself. We suggested it acts as a feedback element, only folding/binding once a sufficient degree of curvature has been achieved, and stabilizes curvature without disrupting the bilayer. However, it remains unclear whether this is unique to RTNLB13 or is conserved across all reticulons—to date, experimental evidence has only been reported for two reticulons. Here we used biophysical methods to characterize a minimal library of putative APH peptides from across the 21 *A. thaliana* isoforms*.* We found that reticulons with the closest evolutionary relationship to RTNLB13 contain curvature-sensing APHs in the same location with sequence conservation. Our data reveal that a more distantly-related branch of reticulons developed a ~ 20-residue linker between the transmembrane domain and APH. This may facilitate functional flexibility as previous studies have linked these isoforms not only to ER remodeling but other cellular activities.

## Introduction

The reticulons (RTNs) are a large family of integral membrane proteins present in all eukaryotes and first discovered in mammalian neurons^1,2^, where they are involved in endocytosis and intracellular transport^3,4^. In addition, they have been linked to apoptosis, axonal growth and regeneration^5,6^. RTNs are also localized to the endoplasmic reticulum (ER), where they are involved in ER shaping from sheets to tubules^7–9^. In many species, deletion of RTNs leads to defects in ER tubule generation, whereas their overexpression increases both ER tubulation and fragmentation^8,10–12^. Abnormal ER morphology caused by mutations in ER-shaping proteins, such as the RTNs, has been implicated in neurological diseases and viral infections^5,13^. ER shaping is therefore not only necessary for maintaining correct ER function, but for the overall health of the cell itself^14,15^.

The function of RTNs is uniquely coupled with generation and recognition of membrane curvature, and the structure of these proteins is exquisitely adapted to this role via a membrane-embedded domain and membrane-peripheral domain acting in concert. The membrane-embedded domain is a highly conserved ~ 200-amino-acid region termed the reticulon homology domain (RHD). This domain is present in all RTNs and is composed of two hydrophobic regions separated by a soluble loop of approximately 60 amino acids^16–18^. These hydrophobic regions are believed to form wedges in the membrane that promote curvature, and this curvature is further stabilized through RTN oligomerization (or scaffolding)^10,19–21^. The membrane-peripheral domain is an amphipathic α-helix (APH) immediately following the RHD which sits on the surface of the membrane at the interface between the polar head-group regions and the hydrophobic core.

Interestingly, despite widely-accepted speculation that the C-terminal APH is conserved across all reticulons, experimental evidence for the presence of this APH has been provided for only two RHD-containing proteins, RTNLB13 (Reticulon-Like Protein subfamily B 13) from *Arabidopsis thaliana* and Yop1p from yeast^22–24^. Brady et al. experimentally demonstrated that the C-term of Yop1p contained a peripheral APH (residues 135–151)^22^ in a region previously shown to be important for protein function^7^. On its own the APH interacted with negatively charged membrane mimetics, whereas in the full-length protein deleting the APH eliminated tubule formation in vitro. The authors hypothesised that it was likely involved in membrane curvature stabilisation, and further suggested it was conserved across all DP1 and RTN family members^22^. Other RHD-containing proteins also rely on C-terminal helices to facilitate generation of highly-curved ER regions; Atg40, an ER-phagy receptor in yeast, was recently shown to contain a short helix adjacent to an Atg8-family interacting motif in the C-term that enhances the binding of this region to Atg8 multimers present on isolation membranes. The resulting super-assembly of Atg40 molecules leads to bending of the membrane via the RHD, and ultimately the formation of an autophagosome^25^. We recently demonstrated that a 14 amino-acid C-terminal amphipathic helix (APH), spanning residues Lys156–Leu169, is a necessary feature for the curvature-generating activity of RTNLB13 in *A. thaliana*^23,24^. RTNLB13 is one of the smallest RTNs in *A. thaliana,* composed almost exclusively of a single RHD, and thus providing an excellent model system for study of membrane remodelling by this family of proteins. Our results suggested that the APH in RTNLB13 stabilizes curved membranes and/or acts as a feedback element once the correct degree of curvature has been achieved by wedging and scaffolding of the hydrophobic RHD^23,24^. This APH inserts a shallow hydrophobic face into lipid packing defects that are a direct result of introducing curvature to a membrane. Notably, the hydrophobic residues that form this binding interface are highly conserved across all *A. thaliana* RTN isoforms^24^.

Given the large degree of variation in the length, sequence composition, and function of RTNs^16,26^, and the difficulty in identifying curvature-responsive APHs from sequence prediction^24^, we suggest that more experimental evidence for the presence of the APH across a number of RTNs is required to establish this emerging model of RTN mechanics. To identify candidate RTNs, we looked again to *A. thaliana*. *A. thaliana* has 21 different RTNLB isoforms, reflecting the much larger RTN gene family in higher plants as compared to mammals. Whilst the rationale for this gene expansion in plants is unknown, it is likely that the various isoforms have cell-specific roles^19,27^.

In this work, we have analysed the sequences of all RTNLB proteins in *A. thaliana* to search for common features such as length, sequence homology and the presence of a putative C-terminal APH. Using the phylogenetic relationship of all 21 RTNLB isoforms, members were grouped into six closely-related clades. Peptides derived from C-terminal sequences predicted to contain an APH, or derived from the C-terminal domain in its entirety, were prepared from members across the clades. Each peptide was exposed to a range of model membranes of varying lipid composition and curvature, and secondary structure was assessed using circular dichroism and solution-state nuclear magnetic resonance (NMR). Our results demonstrate that the C-terminal APH is conserved across all *A. thaliana* RTNLBs known to be involved in ER remodelling, although the location of the APH within the C-terminus appears to have shifted later in evolution. We also highlight key properties these curvature-sensing APHs share despite the fact that the sequence is not strictly conserved. This work adds much needed experimental evidence to speculation that a separate curvature-sensing module, namely an APH, is a conserved feature of all membrane-shaping RTNs. More broadly, this work could direct engineering of existing RTNs or other integral membrane proteins to target curved biological membranes or design of peptide-based therapeutics with curvature-sensing functionality.

## Results

### Phylogenetic, functional, and sequence analysis of RTN isoforms in A. thaliana identifies minimal library of putative RTN APH regions for study

**Figure 1:**
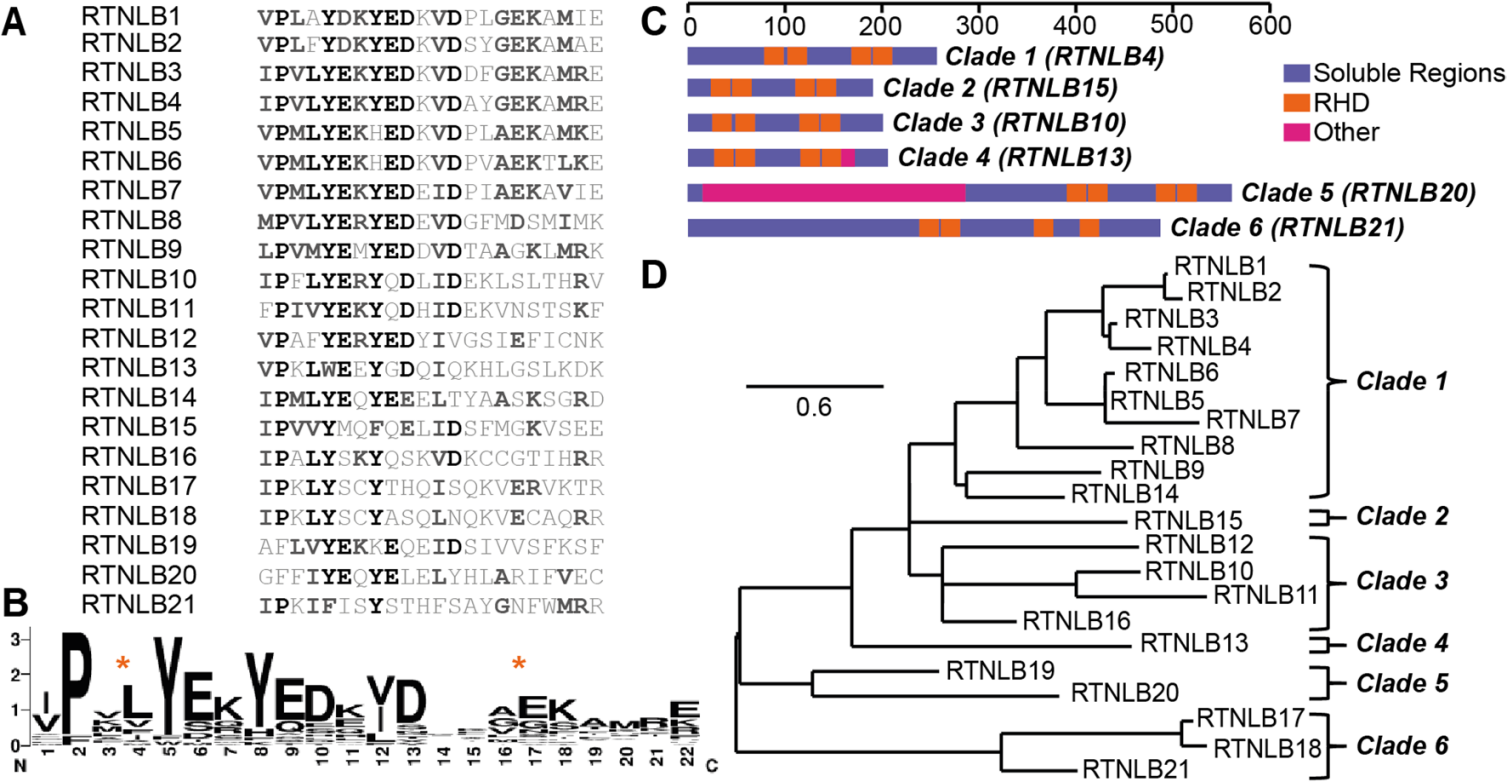
(**A**) Sequence alignment of RTNLB13 APH region of all reticulon-like (RTNLB) protein isoforms found in *A. thaliana*, listed in order from RTNLB1-RTNLB21. Highly conserved residues are highlighted in black, with the shading of the residues decreasing with decreasing conservation. (**B**) Sequence logo to indicate the degree of conservation of the RNLB13 APH-region amongst all RTNLB isoforms in *A. thaliana*. (**C**) Predicted topologies of representative RTNLB isoforms from each clade. The bar at the top refers to number of amino-acids. Each reticulon contains the RHD in orange and the N-terminus, C-terminus and loop regions in violet. Other regions of functionality for specific RTNs are highlighted in magenta, including the APH in RTNLB13 and the 3 beta-hydroxysteroid-dehydrogenase/decarboxylase domain in RTNLB20. (**D**) Phylogenetic analysis of all 21 RTNLB isoforms in *A. thaliana.* The isoforms were then split into six clades based on their evolutionary relationships.

As demonstrated previously^24^, a multiple sequence alignment of all RTN isoforms in *A. thaliana* revealed that the APH region in RTNLB13 was highly conserved across the family (Fig. 1A). Pairwise sequence alignment of this region in all isoforms against RTNLB13 using EMBOSS Stretcher^28^ yielded an average sequence similarity of 51%. Several of the hydrophobic residues that have already been identified as important for the interaction of the APH in RTNLB13 with highly-curved membranes were also the most highly conserved (Fig. 1B)^24^. Here we carried out a phylogenetic analysis of the 21 RTN isoforms in *A. thaliana* in order to group the isoforms according to their evolutionary relationship. As shown in Fig. 1D, RTNLB1-16 are clearly differentiated from RTNLB17-21. When one interrogates the sequences of these proteins, as shown in Fig. 1C, the most recognizable difference between these two groups is the increased length of the N-terminal domain in RTNLB17-21 (179–390 amino acids) as compared to RTNLB1-16 (23–99 amino acids)^29^.

After careful consideration of the evolutionary relationships revealed via the phylogenetic analysis shown in Fig. 1D, we decided to segregate the 21 isoforms into six clades (denoted here as Clades 1–6) to identify a minimal library of RTNs for further study. RTNLB1-16 were split into four clades: RTNLB1-9 and 14 (Clade 1); RTNLB15 (Clade 2); RTNLB10-12 and 16 (Clade 3); and RTNLB13 (Clade 4). Isoforms within these groups, namely RTNLB1-4 and 13, have been previously reported to share the same topology and general properties^10^, and direct ER shaping and membrane remodelling. In addition to ER shaping, RTNLB1-4 and 8 have been implicated in plant–microbe interactions via interactions with the *Agrobacterium tumefaciens* VirB2 protein^30,31^, and RTNLB3 and 6 have been localised to the primary plasmodesmata at cytokinesis, indicating that they may be involved in the formation of the desmotubule^32^.

RTNLB17-21 were split into two clades, RTNLB19-20 (Clade 5 in Fig. 1D) and RTNLB17, 18 and 21 (Clade 6), described by Nziengui et al. previously^27^. As well as containing N-terminal domains that are two–tenfold longer than those of RTNLB1-16, and which have been speculated to encode additional protein functionality^29,33^, it has been shown that isoforms in these groups (RTNLB19 and 20) do not constrict ER tubules when overexpressed and are not involved in ER shaping^29^. The same team suggested that the lack of tubule-forming ability may be due to the absence of an APH in the C-terminus of RTNLB20, which has a higher percentage of hydrophobic residues in comparison to RTNLB13. Instead, it was shown that RTNLB19 and 20 are involved in sterol biosynthesis lipid regulation^29^, and contain a conserved sterol dehydrogenase domain at the N-terminus^33,34^. Although no clear role for the extended N-term in members of Clade 6 has yet been identified, Kreichbaumer et al. did suggest that RTNLB17, 18 and 21 could assist in tethering molecules to the ER membrane via the membrane-embedded RHD and protein–protein interactions in the N-terminus, as has been demonstrated for yeast reticulons and dynamin-related GTPases^27,29,35,36^. The phylogenetic tree in Fig. 1D suggests that the reticulon “tethers” differentiated from the “structural” reticulons early during evolution^36^.

**Figure 2:**
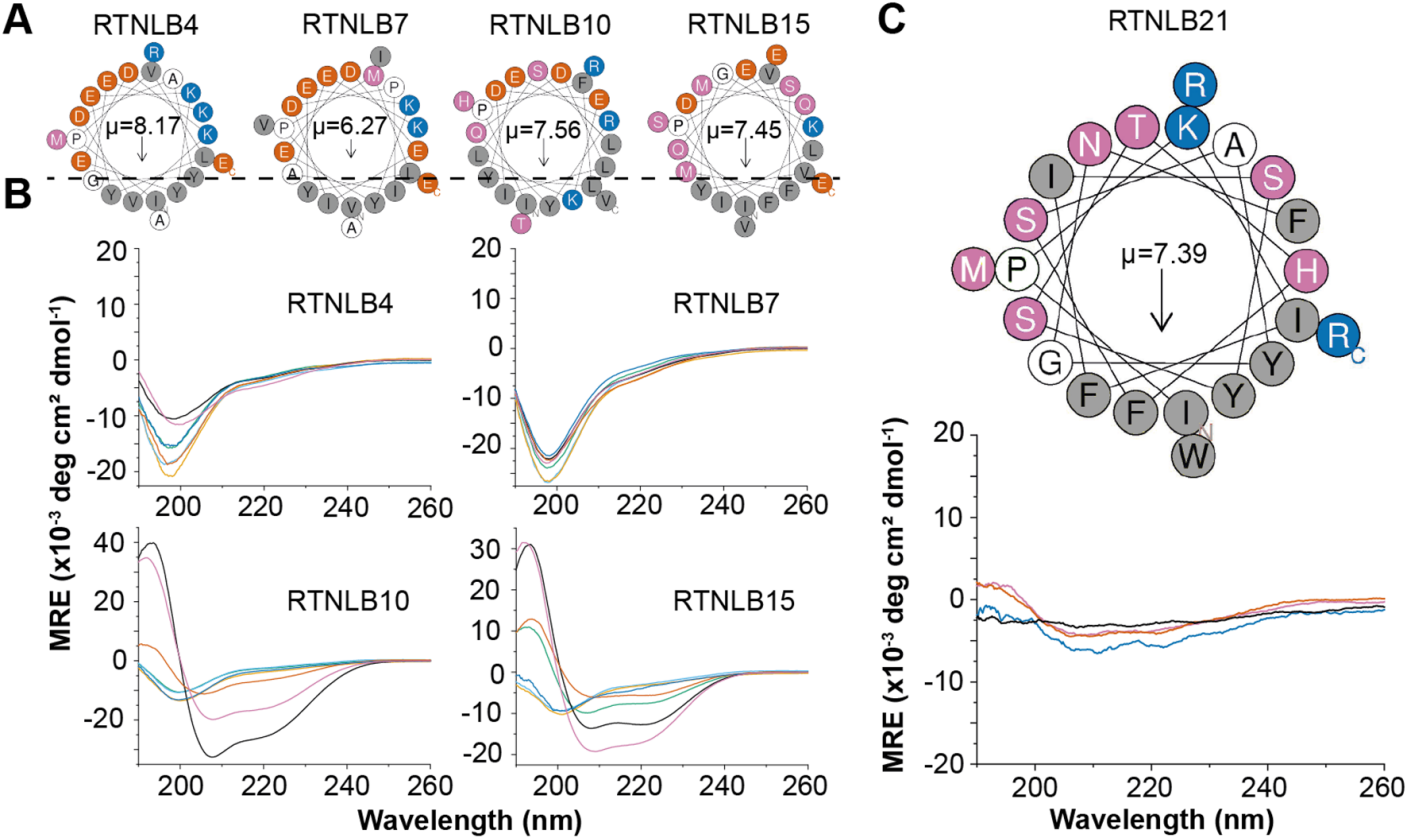
(**A**) Helical wheel plots of the four representative RTNLB peptides chosen for characterization. Each fits the requirements for an APH with distinct hydrophobic and polar faces, and high hydrophobic moments (μ). Hydrophobic residues are shown in grey, acidic residues in red–orange, basic residues in blue, polar residues in purple and other residues in white. (**B**) CD spectra of the four peptides (~ 50 µM) in phosphate buffer and a range of model membranes of different diameters. Specifically, measurements were made in DPC micelles (black, 3.7 nm), LMPG micelles (purple, 4.2 nm), DMPC vesicles (blue, 75 nm), DHPC/DMPC bicelles q = 0.25 (red–orange, 18 nm), and 60:1:15 DMPC:DMPG:DHPC vesicles with diameters of 22 nm (green**)**, 42 nm (sky-blue**)**, and 83 nm (orange). All CD data are given in units of mean residue ellipticity (MRE, deg cm^2^/dmol) and the diameters of the membrane mimetics were estimated using DLS. (**C**) Helical wheel plot of the RTNLB21 peptide and CD spectra of this peptide (17 µM) in buffer (black), DPC (purple), LMPG (red–orange) and DHPC/DMPC bicelles q = 0.25 (blue).

Given that RTNLB19 and 20 have been shown to have no role in constriction of ER tubules and ER shaping^29^ and instead have been implicated in other processes, these isoforms were omitted from our minimal library interrogating conservation of a C-terminal APH. Representative RTNs from Clades 1–3 and Clade 6 were selected, and 22-residue peptides corresponding to the putative APH region, as indicated from multiple sequence alignment (Fig. 1A), were synthesized for comparison to previous results for RTNLB13^24^ (Clade 4). The identity of each representative RTN and the APH peptide sequences are given in Table 1. All five of these peptides fit the requirements for an APH; when plotted on a helical wheel each peptide contains a polar/charged helical face and a hydrophobic face that yields a high hydrophobic moment (Fig. 2). These peptides, in combination with the RTNLB13 APH peptide reported previously^24^, represent a minimal library of RTN APH constructs that would allow us to understand whether or not the APH module is conserved throughout *A. thaliana* RTNs involved in ER shaping and, if so, whether the emerging properties that define such curvature sensing regions are retained.

**Table 1:** Phylogenetic clades of *A. thaliana* RTNLB isoforms, individual members in each clade, and representative members selected.

Clade	Members	Rep. RTN	Putative APH sequence	Residues	MW
1	1–9, 14	4	IPVLYEKYEDKVDAYGEKAMRE	206–227	2647.00
		7	VPMLYEKYEDEIDPIAEKAVIE	205–226	2594.96
2	15	15	IPVVYMQFQELIDSFMGKVSEE	148–169	2490.00
3	10–12,16	10	IPFLYERYQDLIDEKLSLTHRV	162–183	2749.16
4	13	13	See Ref. ^23^		
5	19–20	n/a	n/a		
6	17–18, 21	21	IPKIFISYSTHFSAYGNFWMRR	370–391	2722.16

Putative APH sequences from each representative member are shown, as well as the corresponding position in the sequence and the theoretical peptide molecular weights.

### RTNLB10 and RTNLB15 contain curvature-sensitive APHs at a location similar to that in RTNLB13, but RTNLB4, RTNLB7 and RTNLB21 do not

The four peptides from Clades 1–3 listed in Table 1 are located at similar positions relative to the RHD in each protein (i.e. immediately following the putative fourth transmembrane domain) and have strong sequence similarities with one another and with the APH in RTNLB13. We have shown that the APH in RTNLB13 folds into an α-helix and binds to membrane bilayers in a curvature-dependent manner^24^, therefore replication of this behavior was investigated here across the library of peptides. Each peptide was exposed to model membranes of increasing curvature, and the resulting impact on secondary structure was measured using circular dichroism (CD) spectroscopy. Figure 2B shows the CD spectra for each peptide from Clades 1–3 in DPC and LMPG detergent micelles, DMPC/DHPC bicelles (q = 0.25), DMPC vesicles, and vesicles of composition 60:1:15 DMPC:DMPG:DHPC prepared with increasing diameter. All hydrodynamic diameters were obtained from dynamic light scattering (DLS) measurements in the absence of peptide and are given in the legend in Fig. 2B. This library of model membranes is a more concise version of that utilized in our recent work^24^ designed to include conditions that most clearly demonstrate the response under investigation. CD spectra indicated that all the peptides in Clades 1–3 were soluble and had no defined structure (random coil) in aqueous buffer (Fig. S1). All peptides were also unstructured when the diameter of the model membrane was > 40 nm (Fig. 2B). These results directly reflect the behavior of the APH in RTNLB13 observed in our previous study.

However, only the putative APH peptides from RTNLB10 and RTNLB15 demonstrated a transition in fold from random coil to α-helix upon exposure to increasingly curved model membranes (Fig. 2B). The effect is more pronounced in RTNLB15, although both regions demonstrate a clear propensity to fold as the model membranes decrease in size (increase in curvature). Conversely, the peptides derived from RTNLB4 and RTNLB7 remain unstructured under all conditions tested (Fig. 2B). This result was unexpected given that these regions contain many of the features one would expect from a curvature sensor, i.e. a high hydrophobic moment and a shallow hydrophobic helical face, and reinforces the challenge that curvature sensing regions are difficult to identify from sequence alone^24^.

The peptide chosen from Clade 6 (RTNLB21) aligns with the APH in RTNLB13 but has less sequence similarity compared to the other selected peptides (Fig. 2C). One obvious difference is that this peptide contains no acidic residues. The RTNLB21 peptide was also considerably less soluble than the others under investigation, and only a very weak CD signal was observed when this peptide was exposed to the typically most-solubilizing membrane mimetics (DPC, LMPG, DHPC/DMPC bicelles) as shown in Fig. 2C. This lack of solubility hindered the acquisition of CD spectra of this peptide with lipid vesicles, and suggests that this region of RTNLB21 has very different chemical properties to those selected from Clades 1–3.

**Figure 3:**
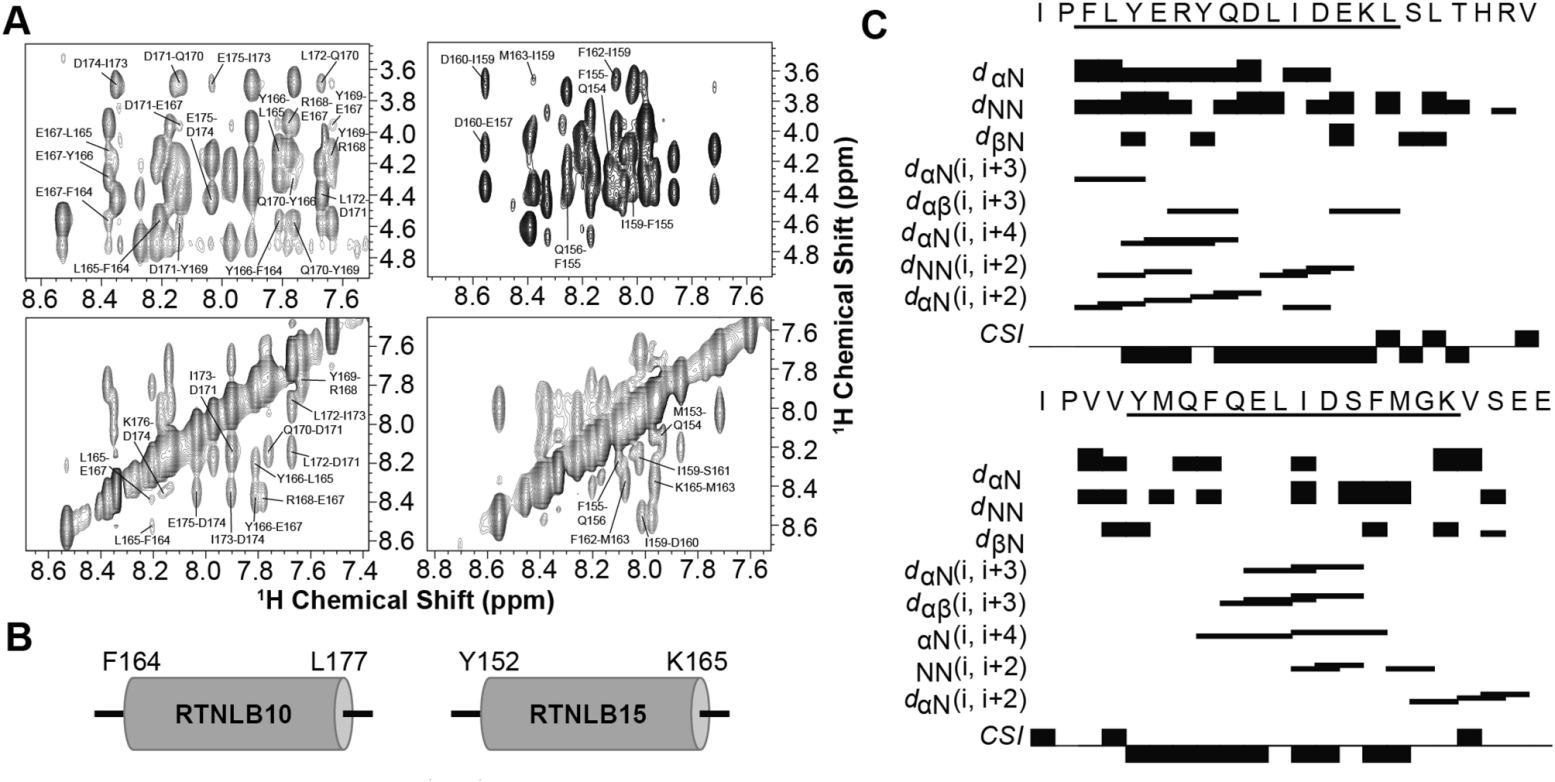
(**A**) Representative ^1^H-^1^H NOESY NMR data for the RTNLB10 (left) and RTNLB15 (right) peptides (~ 1 mM) solubilized in 50 mM DPC-d_38_ at a mixing time of 90 ms. The top panels show the fingerprint regions with characteristic α-helical NOEs labelled (i.e. H_α_ of residue *i*, NH of residues *i* + *1, 3, 4*). The bottom panels show the region of the spectra where backbone amide NOEs are found, also characteristic for α-helices (i.e. NH of residue *i*, NH of residues *i* + 1, 2). (**B**) Schematic showing the NMR-identified locations of the APH regions in RTNLB10 and RTNLB15. (**C**) Survey of NMR-derived sequential backbone NOE connectivities for RTNLB10 (Top) and RTNLB15 (Bottom) peptides solubilized in 50 mM DPC-d_38_, classified as strong, weak, or absent by the thickness (or absence) of a bar connecting the residues involved. The non-sequential connectivities listed (i.e*. i, i* + *n*) are unique to helices, and were used alongside chemical shift index analyses (CSI) to localize the amphipathic helices to those residues underlined in the sequences.

For the two peptides that demonstrated curvature-responsive folding, the same solution-state NMR approach we applied previously^24^ was used to map the α-helices within the sequences. ^1^H-^1^H total correlation spectroscopy (TOCSY) and ^1^H-^1^H nuclear Overhauser spectroscopy (NOESY) spectra were obtained to sequentially assign both peptides (see Fig. 3A, Tables S1 and S2 for assignments and Figs. S2 and S3 for full spectra) in the presence of deuterated DPC micelles, a condition which resulted in significant helical content for both peptides. 83% of the ^1^Hs in the RTNLB10 peptide and 87% of the ^1^Hs in the RTNLB15 peptide were assigned. Long-range backbone (*i*, *i* + *n*) H_N_, Hα and Hβ NOEs indicative of α-helix formation were identified and, together with chemical shift index analysis^37^ of the ^1^Hα chemical shifts (see Fig. 3B), were used to map the α-helical region in RTNLB10 to a 14 amino acid stretch between Phe164–Leu177, and similarly to a region of the same length in RTNLB15, corresponding to Tyr152–Lys165 (Fig. 3B, C). Both of these regions incorporate the most highly conserved residues in this region of the protein, as shown in the sequence logo in Fig. 1B, including the completely conserved aromatic residues.

### The C-terminal domains in RTNLB4 and RTNLB7 contain an APH approximately 20 residues from the putative fourth transmembrane domain

**Figure 4:**
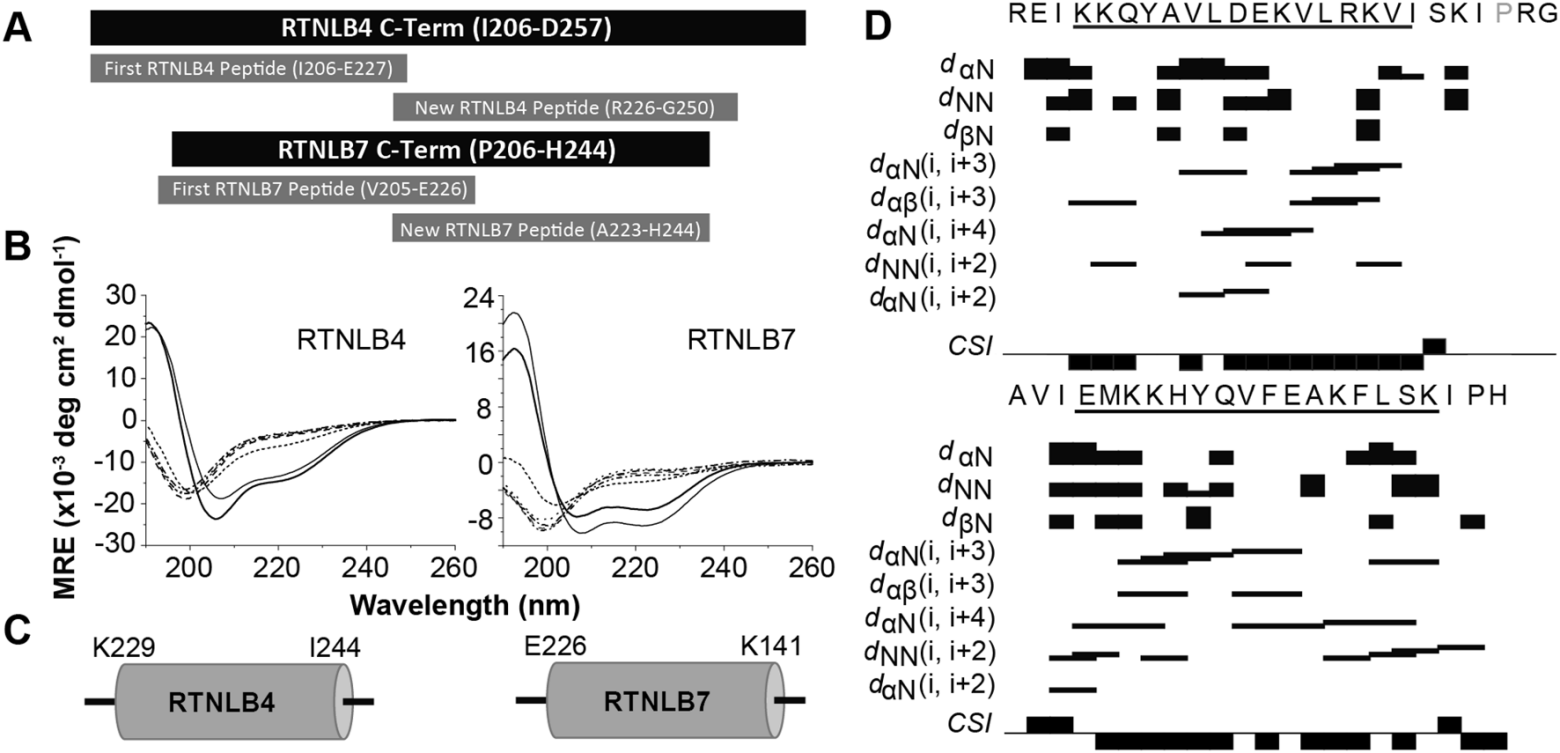
(**A**) Schematic to show the locations of additional RTNLB4 and RTNLB7 peptides further along the C-termini of these two proteins. (**B**) CD spectra of the RTNLB4 (left) and RTNLB7 (right) peptides (~ 50 µM) in phosphate buffer and a range of model membranes: DPC micelles ( **—** , 3.7 nm), LMPG micelles ( — , 4.2 nm), DMPC vesicles ( · · · , 75 nm), DHPC/DMPC bicelles q = 0.25 (- - -, 18 nm), and 60:1:15 DMPC:DMPG:DHPC vesicles with diameters of 22 nm ( **– – – )**, 42 nm ( **– · – )**, and 83 nm (-**· ·-).** All CD data are given in units of mean residue ellipticity (MRE, deg cm^2^/dmol) and the diameters of model membranes were estimated using DLS. (**C**) Schematic showing the NMR-identified locations of the APH regions in RTNLB4 and RTNLB7. (**D**) Survey of NMR-derived sequential backbone NOE connectivities for RTNLB4 and RTNLB7 peptides solubilized in 50 mM DPC-d_38_, classified as strong, weak, or absent by the thickness (or absence) of a bar connecting the residues involved. The non-sequential connectivities listed (i.e*. i, i* + *n*) are unique to helices, and were used alongside chemical shift index analyses (CSI) to localize the amphipathic helices to those residues underlined in the sequences.

The absence of an α-helix in the RTNLB4, RTNLB7, and RTNLB21-derived peptides did not rule out the possibility that an APH module was present at another location in their C-terminal domains. Given that curvature sensors are difficult to identify from sequence, we decided to investigate the entire C-terminus of RTNLB7 as this was the shortest of the three. A 39-residue peptide corresponding to the entire C-Terminal region of RTNLB7 (Fig. 4A, R7-CTerm, residues 206–244) was synthesized and purified. CD data were collected for this peptide in a range of solution conditions and demonstrated generation of α-helical secondary structure in this larger peptide upon exposure to detergent micelles and DMPC/DHPC bicelles, but not in DMPC or 60:1:15 DMPC:DMPG:DHPC vesicles (Fig. S4). The limited solubility of this peptide precluded high-resolution NMR measurements, as concentrations required for NMR were not attainable, and the location of the helical region could not be defined.

An alternative approach was adopted in which shorter peptides corresponding to the extreme C-termini of RTNLB4 and 7 were synthesized, purified and reconstituted into our set of model membranes. These peptides overlapped with the original sequences (see Fig. 4A), covered the majority of the remaining C-termini, and contained a region of sequence predicted to fold into an amphipathic helix (as evaluated using Heliquest^38^). Design of such a peptide for RTNLB21, however, was not possible. Our original RTNLB21 peptide was designed to follow the putative fourth transmembrane domain of the RHD, however it has been speculated in the literature that members of Clade 6 contain an additional membrane-spanning region within their C-termini^27^. Indeed, topology prediction of RTNLB21 using TOPCONS^39^ suggests that this protein contains a fifth transmembrane domain only 13 residues from our peptide between residues ~ 405–426. The C-terminus beyond this putative fifth transmembrane domain is highly polar along its entire length, enriched in acidic residues, and yields no potential APH regions which to target. Therefore, no additional peptide was investigated for RTNLB21.

**Figure 5:**
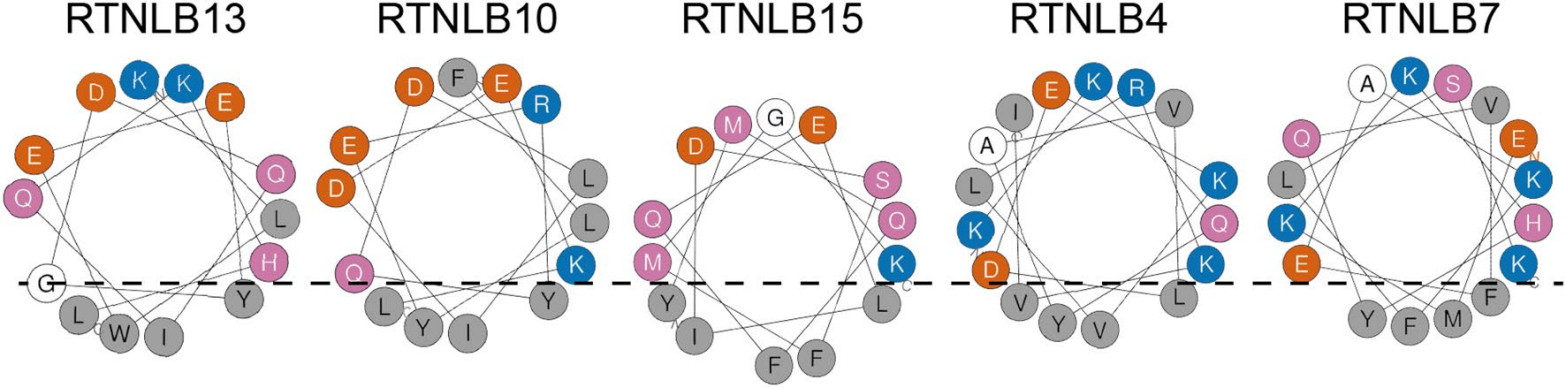
Helical wheel representations of the RTNLB13 APH and the APHs we have characterized in RTNLB10, 15, 4 and 7. Each displays the characteristic shallow hydrophobic face that restricts how far they can penetrate into a membrane in order to avoid bilayer disruption. Hydrophobic residues are shaded grey, polar residues colored purple, acidic residues colored blue, basic residues colored red–orange and other residues are unshaded.

The new RTNLB4 peptide (residues 226–250) and RTNLB7 peptide (residues 223–244) yielded α-helical secondary structure (see CD spectra in Fig. 4B) in the presence of DPC, LMPG and q = 0.25 DMPC/DHPC bicelles while remaining unstructured in DMPC and 60:1:15 DMPC:DMPG:DHPC vesicles of different sizes. Solution-state NMR was used to assign 78% of the ^1^Hs in the DPC-solubilized RTNLB4 peptide (Table S3 and 86% of the ^1^Hs in the RTNLB7 peptide (Table S4), and chemical shift and NOE data were used to map the location of the helical regions in both peptides (Fig. 4C, D). In the case of RTNLB4, a C-terminal APH is present between residues Glu226–Ser240 and in RTNLB7 a C-terminal APH is present between residues Lys229–Ile244_._ These sequences are plotted on a helical wheel diagram in Fig. 5 to demonstrate that both regions fit the description of an APH, and in both cases the APH is 15–16 residues in length and 20–23 residues from the reticulon homology domain.

## Discussion

**Figure 6:**
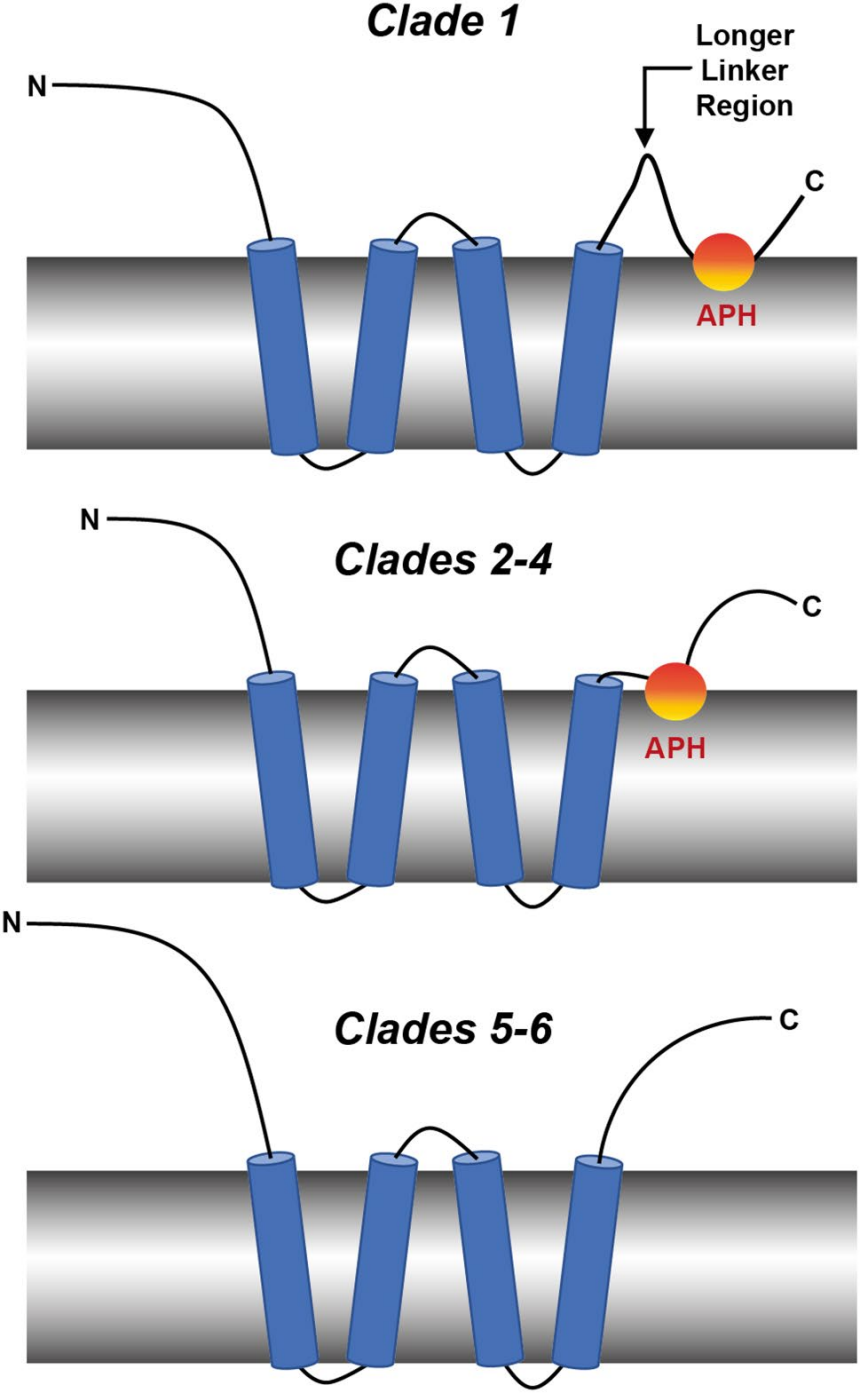
Schematic summarizing the structural features of the RTNLB proteins in *A. thaliana* proposed from the results of this work. For RTNLBs in Clade 1, a curvature-responsive APH is located approximately 20 residues from the membrane-spanning RHD. This APH is located much closer to the membrane-spanning RHD in Clades 2–4, and we (and others) propose this APH may be missing in Clades 5–6.

Recent studies of a yeast and a plant reticulon suggest that an APH following the reticulon homology domain is critical for membrane remodeling and ER shaping by this family of proteins^22–24^, however experimental data has only been reported for two proteins, RTNLB13^23,24^ and Yop1p^22^. Here we have examined all 21 RTN isoforms from *A. thaliana* to create a library of peptides in order to establish whether this feature is indeed conserved, or if the APH in RTNLB13 is unique. We have summarized our findings in a structural schematic shown in Fig. 6. Inspection of the phylogenetic tree in Fig. 1A revealed that all *A. thaliana* RTNLB isoforms share a common ancestor that most likely contained the RHD. RTNLB17-21 branch away from RTNLB1-16 early in evolution, and the RHD in these proteins is thought to simply tether them to the ER for other functions^27,29,35,40^ as these proteins are *not* believed to be involved in shaping the ER. Therefore, isoforms in these two clades are proposed to not contain a curvature-sensitive APH in their C-termini (Fig. 6, bottom panel) and may instead contain other features such as an additional transmembrane domain^27^.

RTNLB1-16 all share a common ancestor that RTNLB17-21 do not, therefore a speciation event could have resulted in ER-shaping functionality. RTNLB13 has the closest evolutionary relationship to this common ancestor and, as we have previously shown^24^, contains an essential APH immediately following the RHD that has likely been inherited (along with curvature-generation function) by all the other RTNs in Clades 1–4. RTNLB15 is most closely related to RTNLB13, and our results show that there is a putative APH in this protein that has a similar location, length, sequence, and curvature sensitivity (> 30 nm diameter) to that in RTNLB13. RTNLB10 is the next most-closely related to RTNLB13 according to the phylogenetic tree shown in Fig. 2A, and again contains an APH with a similar location, length and sequence, but this isoform appears to have a different curvature sensitivity (only folding in membrane mimetics of diameter < 20 nm). This difference in curvature-sensitivity could be within the error of the measurements, or may be attributed to the fact that ER tubules are highly dynamic, flexible and undergo constant remodeling^41^, and therefore different RTN isoforms may be active at different stages^30^. Additionally, the ER morphology varies between cells so these proteins could have cell-specific roles within *A. thaliana*. Overall, these results allow us to say with some confidence that the location of the C-terminal APH is likely conserved throughout *A. thaliana* RTNLB isoforms RTNLB10-13 and RTNLB15-16 (Fig. 6, center panel).

RTNLB4 and 7 were selected as representatives of Clade 1 (Fig. 2A), and both proteins did not contain putative APHs in the same location as those in RTNLB10, 13 and 15; the APHs were instead located ~ 20 residues from the RHD. Proteins with multiple domains often contain short linker regions that are essential in maintaining inter-domain interactions or to facilitate the independent behavior of the two functional domains^42^. While linker regions are challenging to predict from sequence^43^, early studies concluded that linkers lack regular secondary structure, are rich in Ala, Pro and charged residues, and exhibit differing levels of flexibility depending on their biological role^44–46^. Both of the linker regions in RTNLB4 and 7 have a higher abundance of negatively charged residues than the equivalent regions of RTNLB10, 13 and 15, especially in regions predicted to lie near lipid head groups (see Fig. 2A), and this may prevent this region from folding into an APH thus shifting it further down the sequence. It is not immediately clear why introducing a longer linker region would be necessary in these isoforms, although members of this group have been shown to localize to other parts of the cell and have functions separate to their roles in ER remodeling, e.g. RTNLB1-4 are thought to also be involved in plant–microbe interactions^30–32^. Additionally, a previously mentioned RTN-like protein, Atg40, contains a short helix in its C-term that strengthens binding of this region to a protein-partner^25^. This particular helix is considerably further along the C-term from the transmembrane domains and is rich in acidic residues; these features likely allow this helix to function independently of the RHD. Taken together, these results suggest that the presence of a C-terminal APH is conserved throughout *A. thaliana* RTNLB1-9 and 14, and that a ~ 20 residue linker between the APH and the RHD was introduced for these isoforms (Fig. 6, top panel).

Consideration of this set of five RTN APH regions (Fig. 5) presents the opportunity to draw comparisons that have not been possible thus far. Our data suggest a range of lipid sensitivities for APH folding across the family of *A. thaliana* isoforms. The APH regions in RTNLB13 and 15 show no sensitivity to lipid composition and readily fold on highly curved model membranes regardless of charge. This was not the case for the APH regions in RTNLB4, 7 and 10. These APHs did not fold in 60:1:15 DMPC:DMPG:DHPC vesicles which we used to access highly curved bilayer structures^47^. Inspection of the structural models shown in Fig. 5 reveals that these three APHs uniquely contain acidic residues at or near the hydrophobic helical face. Charge-charge repulsion between these acidic residues and negatively-charged PG head groups may be sufficient to destabilize helix formation. Notably, RTNLB13 and 15 contain no acidic residues at this position and experience no charge-dependent destabilization. PG is not a typical constituent of the ER membrane; It is mostly found in the inner mitochondrial membrane where it can also be synthesized^12^ and in bacterial membranes^48^. An obvious next step would be measurement of APH folding in the presence of tiny vesicles containing no PG lipids, however as reported by us previously^24^ this has not yet been possible due to vesicle instability in the absence of negatively-charged PG^47^. Work is ongoing to better understand the impact of lipid composition on these regions. This work once again highlights how difficult it can be to identify curvature-sensing helical motifs by simply examining amino acid sequences. However, despite the differences in the sequence and location, all five of these APHs share key features that we identified previously as directing curvature-sensitivity and affinity for ER membranes^24^: they are between 14 and 16 residues in length; they contain shallow hydrophobic faces composed of 4–5 residues (Fig. 5); and there is no requirement for the presence of charge for association (in some cases this can prevent interaction). This work provides much needed experimental evidence supporting the presence of a conserved APH module critical for membrane remodeling by RTNs, and more broadly reveals shared features that will facilitate the recognition of other membrane curvature sensors in the future.

## Methods

### Sequence alignment, phylogenetic analysis and topology prediction

Sequences for all reticulon-like (RTNLB) protein isoforms found in *A. thaliana* were obtained from UniProt^49^, sequence alignment was carried out using T-Coffee and formatted using Boxshade^28^. A Pairwise Sequence alignment of all the RTNLB isoforms in *A. thaliana* against RTNLB13 was performed using Emboss Stretcher^28^. Sequence logo indicating the degree of conservation was generated using WebLogo 3^50^. The phylogenetic tree was rendered using the Phylogeny.fr “One Click” mode^51^, with sequences obtained from UniProt^49^. Protein topology was predicted using TOPCONS^39^ and helical wheels were obtained from Heliquest^38^.

### Synthesis and purification of RTNLB APH peptides

Five peptides corresponding to putative APH regions of *A. thaliana* RTNLB4, 7, 10, 15 and 21 were synthesized using F-moc chemistry and purified to 95% purity at Insight Biotechnology Limited (Wembley, UK). The identity, amino acid sequence, and molecular weights for these peptides are shown in Table 1. Additionally, a peptide containing the entire putative C-terminal domain of RTNLB7 (residues 206–244; PMLYEKYEDEIDPIAEKAVIEMK KHYQVFEAKFLSKIPH; M_W_ 4709.50) and the C-terminal 22 residues of this region (residues 223–244; AVIEMKKHYQVFEAKFLSKIPH; M_W_ 2644.17) were prepared. A further peptide derived from the C-term of RTNLB4 (residues 226–250; REIKKQYAVLDEKVLRKVISKIPRG; M_W_ 2967.59) was also synthesized. The purity of all peptides studied here was confirmed by HPLC and electrospray ionization time-of-flight mass spectroscopy (ESI-TOF–MS microTOF, Bruker). All peptides were stored at − 20 °C as lyophilized powders until use.

### Bicelle and vesicle preparation

All lipids and detergents were obtained from Avanti Polar Lipids (Alabaster, AL) and used without any additional purification. Bicelles with a *q* value of 0.25 (*q* = [DMPC]/[DHPC]) and a total lipid concentration of 150 mM were generated using 1,2-dimyristoyl-*sn*-glycero-3-phosphocholine (DMPC) and 1,2-dihexanoyl-*sn*-glycero-3-phosphocholine (DHPC). An appropriate amount of DMPC was mixed with 25 mM sodium phosphate buffer, pH 6.8, followed by continuously vortexing and centrifuging the mixture until a homogeneous slurry formed, and adding a suitable amount of a 400 mM DHPC solution. This mixture was then subjected to several cycles of centrifugation and vortexing until a clear non-viscous solution was attained. DMPC vesicles were prepared by dissolving an appropriate amount of lipid in 3:1 chloroform:methanol to reach a concentration of 10 mg/mL, and subsequently dried via a rotary evaporator to acquire a thin lipid film. The film was then reconstituted in 25 mM sodium phosphate buffer, pH 6.8, to a final concentration of 3.3 mg/mL and treated to four freeze–thaw cycles, followed by sonication for 2 min. Prior to use, the DMPC vesicles were extruded through polycarbonate membranes (Avanti Polar Lipids) with pore diameters of 100 nm. Vesicles of diameter < 30 nm containing DMPC, DHPC and 1,2-dimyristoyl-*sn*-glycero-3-phospho-(1-*rac*-glycerol) (DMPG) were prepared by adapting a protocol previously described by Yue et al*.*^47^ Briefly, two solutions containing 4:1 DMPC:DHPC and 60:2:15 DMPC:DMPG:DHPC and a total lipid concentration of 50 mg/mL each, were repeatedly vortexed and temperature cycled from 4 to 50 °C in order to dissolve the lipids and then combined to achieve a final composition of 60:1:15 DMPC:DMPG:DHPC. This mixture was then allowed to equilibrate at 4 °C for 24 h before diluting to a final total lipid concentration of 10 mg/mL and subjecting to one freeze–thaw cycle. Vesicles with the same composition but larger sizes were allowed to equilibrate for up to one week instead of 24 h and extruded through either a 50 or a 100 nm polycarbonate membrane before use. All reported diameters were verified via dynamic light scattering.

### Circular dichroism

Samples for CD measurements contained peptide at a constant concentration of ~ 50 mM, as determined by measuring absorbance at 280 nm (A_280_), in 25 mM sodium phosphate buffer at pH 6.8, and either no membrane mimetic or a membrane mimetic at an appropriate concentration. CD spectra were recorded using a J-1500 spectropolarimeter supplied with a Peltier thermally controlled cuvette holder (Jasco UK, Great Dunmow, UK) and a 1.0 mm pathlength quartz cuvette (Starna, Optiglass Ltd, Hainault, UK). Spectra were measured at 37 °C between 190 and 300 nm, with a bandwidth of 2.0 nm, step resolution of 0.2 nm, scanning speed of 200 nm/min, response time of 1 s and averaged from 16 individual scans after subtraction of the buffer spectrum. To normalize to protein concentration, the machine units of mdeg were converted to mean residue ellipticity. An estimate of reproducibility in our CD measurements is provided in Fig. S5, which contains representative error analysis from three technical repeats for select peptides.

### Dynamic light scattering

The hydrodynamic diameter of each membrane mimetic was estimated by dynamic light scattering (DLS) using a Zetasizer Nano-series instrument (Malvern Instruments, UK) at room temperature, with 1 cm path length UV-transparent disposable cuvettes. Mimetic samples were diluted to a concentration of 0.06 mg/ml with 25 mM sodium phosphate buffer at pH 6.8. Each sample was measured six times and each measurement consisted of sixteen accumulations, and were recorded after 300 s equilibration time. To determine the error in these measurements the standard deviation was calculated for each mimetic; DPC (3.7 ± 0.72 nm); LMPG (4.2 ± 0.67 nm); DMPC/DHPC bicelles (18 ± 3.63 nm); DMPC (75 ± 11.1 nm); 60:1:15 DMPC:DMPG:DHPC (22 ± 4.1 nm); 60:1:15 DMPC:DMPG:DHPC (42 ± 6.5 nm); 60:1:15 DMPC:DMPG:DHPC (83 ± 8.4 nm). The Malvern Zetasizer software was utilized for processing data and afterwards exported as intensity and number distributions.

### Nuclear magnetic resonance experiments

Samples for ^1^H-^1^H TOCSY and NOESY NMR experiments contained peptides at a concentration in the range of 1–1.5 mM, as determined by measuring absorbance at 280 nm (A_280_), in 25 mM sodium phosphate buffer, pH 6.8, with 90% H_2_O, 10% D_2_O and 50 mM DPC-d_38_. NMR experiments were carried out on an 700 MHz Avance spectrometer (Bruker Biospin, UK) equipped with a triple resonance inverse cryoprobe with Z-gradients using 3 mm diameter NMR tubes (Bruker, Germany). Spectra were obtained at 37 °C with 4096 × 256 data points, 14 × 14 ppm spectral windows, 32 scans, and referenced to residual water. Mixing times for TOCSY and NOESY ranged from 70 to 140 ms and 90 to 200 ms, respectively. Topspin 3.2 (Bruker Biospin, UK) was used for processing spectra and CcpNmr Analysis V2^52^ for assignment. Hα chemical shift data from 2 to 3 separate TOCSY spectra were used to estimate error in chemical shift. We find all Hα chemical shifts, which are crucial in mapping regions of helicity using CSI methods, are accurate within 0.005 ppm.

## Supplementary Information

Supplementary Information 1.
