# Supplementary material for: Curvature sensing amphipathic helix in the C-terminus of RTNLB13 is conserved in all endoplasmic reticulum shaping reticulons in Arabidopsis thaliana
Source: Sci Rep. 2021 Mar 18;11:6326. doi: 10.1038/s41598-021-85866-3 (PMC7973432; doi:10.1038/s41598-021-85866-3)
Supplement: Supplementary file 1 — Supplementary Information 1. [file 41598_2021_85866_MOESM1_ESM.docx]

**Curvature Sensing Amphipathic Helix in the C-terminus of RTNLB13 is conserved in all endoplasmic reticulum shaping reticulons in Arabidopsis thaliana**

Rhiannon L. Brooks^1,2^, Chandni S. Mistry^2^ and Ann M. Dixon^2,*^

^1^ MAS Centre for Doctoral Training, and ^2^ Department of Chemistry, University of Warwick, Coventry, CV4 7AL, UK.

*To whom correspondence should be addressed: Dr Ann Dixon, Department of Chemistry, University of Warwick, Coventry, CV4 7AL, UK, Telephone: +44 2476 150037; FAX: +44 2476 524112; email: [ann.dixon@warwick.ac.uk](mailto:ann.dixon@warwick.ac.uk)

**SUPLEMENTARY INFORMATION FIGURES**

**Figure S1.** *Related to Figure 2B.* Circular dichroism spectra of RTNLB4, 7, 10 and 15 APH peptides (~50 µM) in phosphate buffer. The peptides all display random coil secondary structure in buffer. All CD data are given in units of mean residue ellipticity (MRE, deg cm^2^/dmol).

**
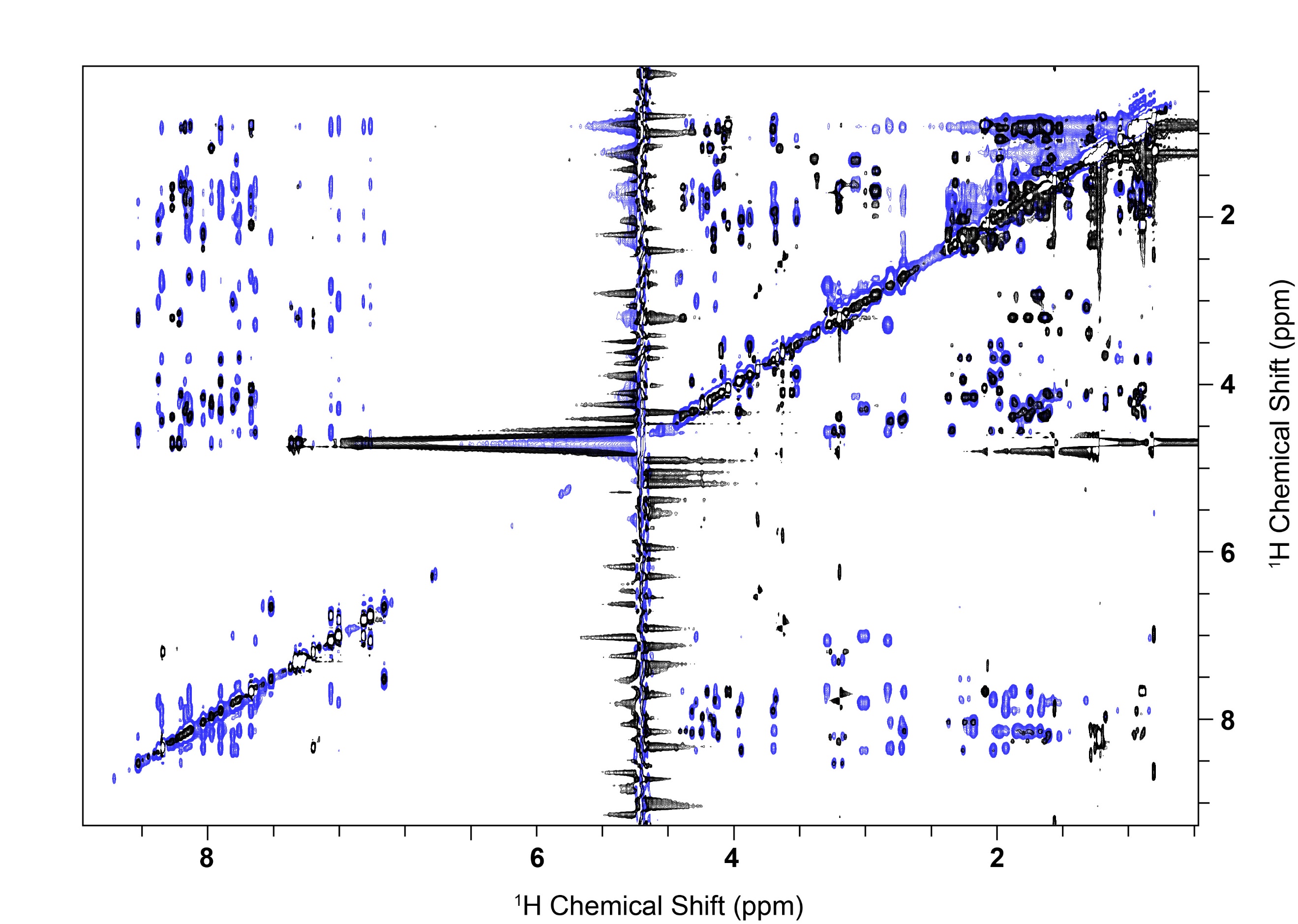
**

**Figure S2.** *Related to Figure 3 and Table S1.* Overlay of ^1^H-^1^H TOCSY (black) and ^1^H-^1^H NOESY (blue) spectra for RTNLB10 peptide (1 mM) in the presence of 50 mM DPC- d_38_ detergent.


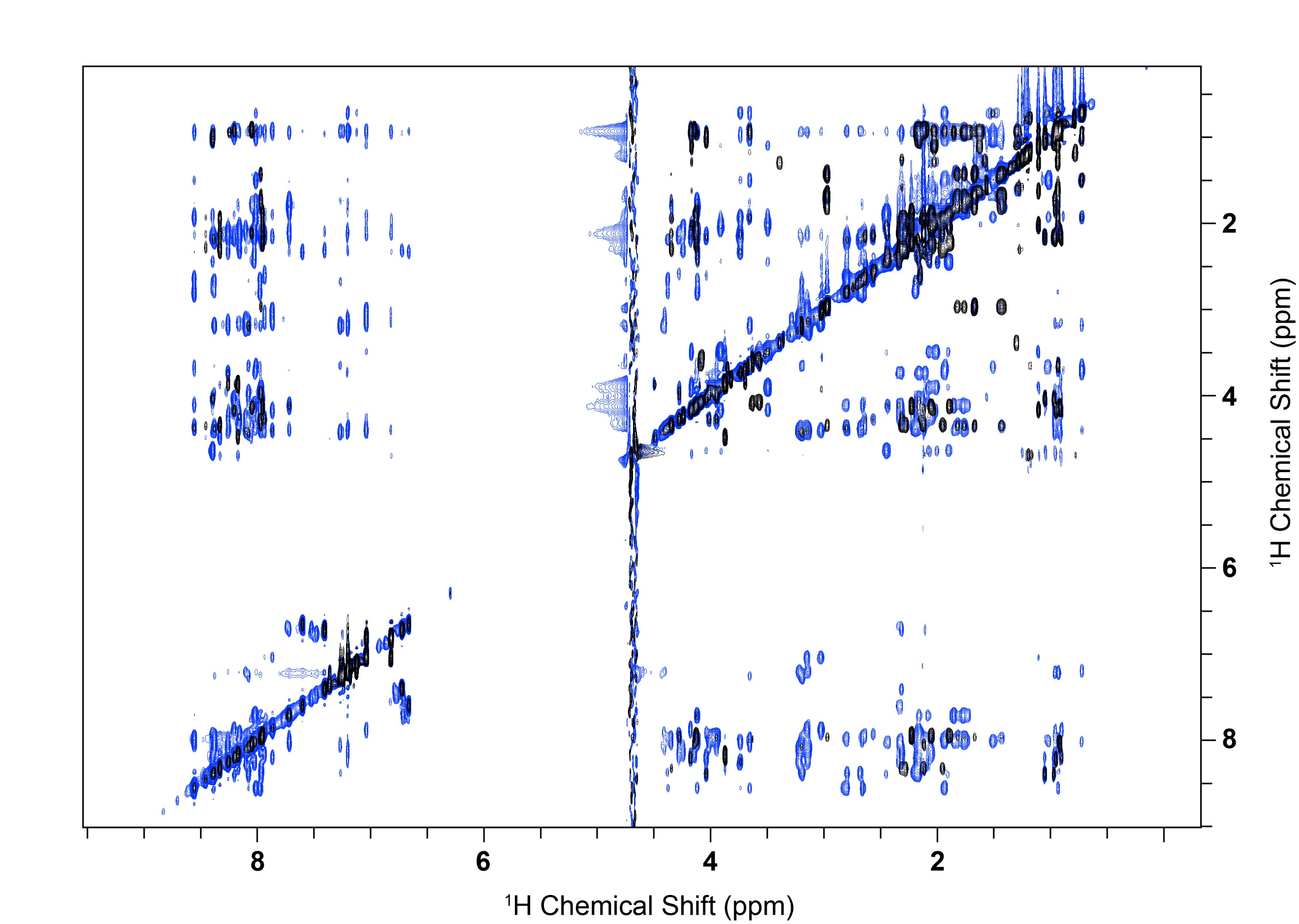


**Figure S3.** *Related to Figure 3 and Table S2.* Overlay of ^1^H-^1^H TOCSY (black) and ^1^H-^1^H NOESY (blue) spectra for RTNLB15 peptide (1.2 mM) in the presence of 50 mM DPC- d_38_ detergent.

**Figure S4.** *Related to Figure 4.* Circular dichroism spectra of the RTNLB7 C-Terminal peptide (~50 µM) in the presence of DPC micelles (black), DMPC/DHPC Bicelles with q = 0.25 (red), DMPC vesicles (blue), 60:2:15 DMPC:DMPG:DHPC 30 nm vesicles (green) and LMPG micelles (purple). All CD data are given in units of mean residue ellipticity (MRE, deg cm^2^/dmol).


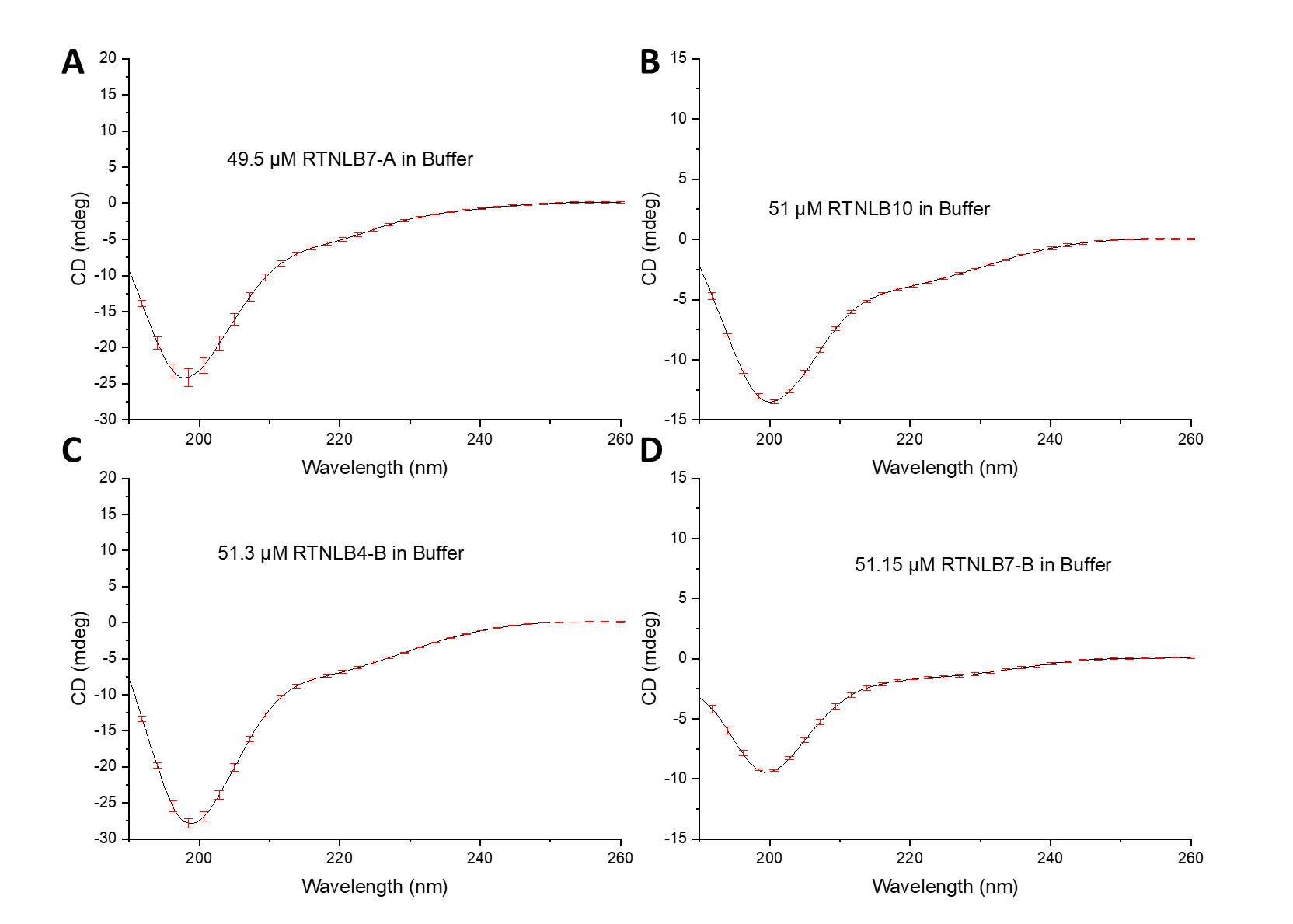


**Figure S5.** *Related to Methods.* Representative CD spectra showing the average spectrum of three technical repeats of **A.** 49.5 µM of the original RTNLB7 sequence. **B.** 51 µM RTNLB10 peptide. **C.** 51.3 µM of the second RTNLB4 peptide. **D.** 51.15 µM of the second RTNLB7 peptide. All peptides were solubilized in 25 mM sodium phosphate buffer at pH 6.8. The error bars show the standard deviation for the CD signal (in mdeg) every 10 nm.

**SUPPLEMENTARY INFORMATION TABLES**

**Table S1.** *Related to Figure 3..* ^1^H NMR assignment of the RTNLB10 peptide (1 mM) in the presence of 50 mM DPC-d_38_ detergent.

| Residue | NH | αH | βH | Others |  |
| --- | --- | --- | --- | --- | --- |
| Ile 162 | 7.66 | 4.05 | 2.10 | γCH_2_  γCH_3_  δCH_3_ | 1.03  0.90 |
| Pro 163 | - | 4.09 | 3.62,  3.57 | γCH_2_  δCH_2_ | 1.98  3.88 |
| Phe 164 | 8.53 | 4.56 | 3.23,  3.16 | 2,6H  3,5H  4H | 7.30  7.43  7.37 |
| Leu 165 | 8.20 | 4.12 | 1.61 | γH  δCH_3_ | 1.52  0.95 |
| Tyr 166 | 7.81 | 4.29 | 3.03,  3.01 | 2,6H  3,5H | 7.01  6.81 |
| Glu 167 | 8.38 | 3.95 | 2.03 | γCH_2_ | 2.26 |
| Arg 168 | 7.78 | 4.14 | 1.66 | γCH_2_  δCH_2_  εNH | 1.32  3.08  7.37 |
| Tyr 169 | 7.64 | 4.58 | 3.29,  2.83 | 2,6H  3,5H | 7.07  6.76 |
| Gln 170 | 7.76 | 3.69 | 2.04,  1.93 | γCH_2_  δNH_2_ | 2.28, 2.22  unassigned |
| Asp 171 | 8.14 | 4.40 | 2.71 | - |  |
| Leu 172 | 7.68 | 4.04 | 2.09 | γH  δCH_3_ | 0.90 |
| Ile 173 | 7.89 | 3.70 | 1.94 | γCH_2_  γCH_3_  δCH_3_ | 1.56  0.94 |
| Asp 174 | 8.35 | 4.44 | 2.81,  2.73 | - |  |
| Glu 175 | 8.03 | 4.15 | 2.25,  2.18 | γCH_2_ | 2.37 |
| Lys 176 | 8.16 | 4.25 | 1.99 | γCH_2_  δCH_2_  εCH_2_  εNH_3_^+^ | 1.46  1.68  2.92  unassigned |
| Leu 177 | 8.13 | 4.38 | 1.83 | γH  δCH_3_ | 1.66  0.90 |
| Ser 178 | 7.91 | 4.32 | 3.96 | − |  |
| Leu 179 | 8.17 | 4.32 | 1.79 | γH  δCH_3_ | 1.62  0.95, 0.90 |
| Thr 180 | 7.98 | 4.24 | 4.18 | γCH_3_ | 1.18 |
| His 181 | 8.22 | 4.71 | 3.27,  3.15 | 2H  4H | 8.34  7.20 |
| Arg 182 | 8.27 | 4.39 | 1.89,  1.77 | γCH_2_  δCH_2_  εNH | 1.64  3.21  7.31 |
| Val 183 | 7.67 | 4.21 | 1.88 | γCH_3_ | 0.98 |

**Table S2.** *Related to Figure 3.* ^1^H NMR assignment of the RTNLB15 peptide (1.2 mM) in the presence of 50 mM DPC-d_38_ detergent.

| Residue | NH | αH | βH | Others |  |
| --- | --- | --- | --- | --- | --- |
| Ile 148 | 7.71 | 4.12 | 1.85 | γCH_2_  γCH_3_  δCH_3_ | 1.78  0.96  0.93 |
| Pro 149 | - | 4.09 | 2.34 | γCH_2_  δCH_2_ | 3.63, 3.58  unassigned |
| Val 150 | 8.39 | 4.04 | 2.17 | γCH_3_ | 1.03, 0.97 |
| Val 151 | 8.20 | 4.18 | 2.17 | γCH_3_ | 0.94, 0.90 |
| Tyr 152 | 7.86 | 4.41 | 3.15,  3.02 | 2,6H  3,5H | 7.04  6.82 |
| Met 153 | 7.94 | 4.28 | 2.15 | γCH_2_  εCH_3_ | 2.64, 2.58  unassigned |
| Gln 154 | 8.15 | 4.24 | 2.07 | γCH_2_  δNH_2_ | 2.32  7.04, 6.64 |
| Phe 155 | 8.10 | 4.40 | 3.16 | 2,6H  3,5H  4H | 7.20  7.26  7.25 |
| Gln 156 | 8.26 | 3.73 | 2.16,  2.12 | γCH_2_  δNH_2_ | 2.33  7.72, 7.36 |
| Glu 157 | 7.95 | 4.12 | 2.05,  1.89 | γCH_2_ | 2.23, 2.15 |
| Leu 158 | 8.25 | 4.19 | 2.12 | γH  δCH_3_ | 0.95 |
| Ile 159 | 8.01 | 3.66 | 1.94 | γCH_2_  γCH_3_  δCH_3_ | 1.51, 1.18  0.94  0.73 |
| Asp 160 | 8.55 | 4.38 | 2.80,  2.65 | - |  |
| Ser 161 | 8.26 | 4.49 | 3.87 | - |  |
| Phe 162 | 8.07 | 4.43 | 3.19 | 2,6H  3,5H  4H | 7.26  unassigned  unassigned |
| Met 163 | 8.38 | 4.38 | 2.20 | γCH_2_  εCH_3_ | 2.80, 2.67  unassigned |
| Gly 164 | 7.98 | 3.96 | - | - |  |
| Lys 165 | 7.97 | 4.34 | 1.80,  1.74 | γCH_2_  δCH_2_  εCH_2_  εNH_3_^+^ | 1.43  1.67  2.97  unassigned |
| Val 166 | 8.05 | 4.15 | 2.44 | γCH_3_ | 0.92 |
| Ser 167 | 8.17 | 4.49 | 3.87 | - |  |
| Glu 168 | 8.33 | 4.34 | 2.12,  1.95 | γCH_2_ | 2.29 |
| Glu 169 | 8.45 | 4.35 | 2.13,  1.94 | γCH_2_ | 2.29 |

**Table S3.** *Related to Figure 4.* ^1^H NMR assignment of the new RTNLB4 peptide (1.2 mM) in the presence of 50 mM DPC-d_38_ detergent.

| Residue | NH | αH | βH | Others |  |
| --- | --- | --- | --- | --- | --- |
| Arg 226 | 7.46 | 4.46 | 1.97 | γCH_2_  δCH_2_  εNH | 1.73  3.22  unassigned |
| Glu 227 | 8.89 | 4.50 | 2.06,  1.96 | γCH_2_ | 2.30, 2.27 |
| Ile 228 | 8.56 | 4.16 | 1.90 | γCH_2_  γCH_3_  δCH_3_ | 1.53, 1.23  0.92  0.88 |
| Lys 229 | 8.42 | 4.25 | 1.82,  1.73 | γCH_2_  δCH_2_  εCH_2_  εNH_3_^+^ | 1.44, 1.40  1.69  2.98  unassigned |
| Lys 230 | 8.24 | 4.22 | 1.74,  1.69 | γCH_2_  δCH_2_  εCH_2_  εNH_3_^+^ | 1.42, 1.38  unassigned  2.98  unassigned |
| Gln 231 | 8.29 | 4.24 | 1.96 | γCH_2_  δNH_2_ | 2.24  unassigned |
| Tyr 232 | 8.05 | 4.51 | 3.09,  2.97 | 2,6H  3,5H | 7.09  6.81 |
| Ala 233 | 8.04 | 4.28 | 1.43 | - |  |
| Val 234 | 7.82 | 4.06 | 2.20 | γCH_3_ | 0.99, 0.96 |
| Leu 235 | 7.79 | 4.32 | 1.74 | γH  δCH_3_ | 1.55  0.92 |
| Asp 236 | 7.72 | 4.65 | 2.91,  2.79 | - |  |
| Glu 237 | 8.91 | 3.94 | unassigned | γCH_2_ | 2.17 |
| Lys 238 | 8.32 | 4.03 | 2.03,  1.84 | γCH_2_  δCH_2_  εCH_2_  εNH_3_^+^ | 1.46  1.74  3.03  unassigned |
| Val 239 | 7.89 | 3.77 | 2.27 | γCH_3_ | 1.14, 1.00 |
| Leu 240 | 7.89 | 4.01 | 1.79 | γH  δCH_3_ | unassigned  unassigned |
| Arg 241 | 7.59 | 3.97 | 1.91 | γCH_2_  δCH_2_  εNH | 1.70  3.22  unassigned |
| Lys 242 | 7.63 | 4.14 | 2.01 | γCH_2_  δCH_2_  εCH_2_  εNH_3_^+^ | 1.58  1.71  2.97  unassigned |
| Val 243 | 8.04 | 3.68 | 2.29 | γCH_3_ | 1.09, 0.95 |
| Ile 244 | 8.15 | 3.80 | 2.00 | γCH_2_  γCH_3_  δCH_3_ | 1.31  0.99  unassigned |
| Ser 245 | 7.43 | 4.70 | 4.09 | - |  |
| Lys 246 | 7.63 | 4.35 | 1.96 | γCH_2_  δCH_2_  εCH_2_  εNH_3_^+^ | unassigned  unassigned  unassigned  unassigned |
| Ile 247 | 7.43 | 4.15 | 1.94 | γCH_2_  γCH_3_  δCH_3_ | 1.67, 1.18  0.98  unassigned |
| Arg 249 | 8.20 | 4.46 | 1.97 | γCH_2_  δCH_2_  εNH | 1.74  3.22  unassigned |
| Gly 250 | 8.27 | 3.95 | - | - |  |

**Table S4.** *Related to Figure 4.* ^1^H NMR assignment of the new RTNLB7 peptide (1.5 mM) in the presence of 50 mM DPC-d_38_ detergent.

| Residue | NH | αH | βH | Others |  |
| --- | --- | --- | --- | --- | --- |
| Ala 223 | 8.27 | 4.31 | 1.37 | - |  |
| Val 224 | 7.66 | 4.31 | 1.78 | γCH_3_ | 1.08, 0.85 |
| Ile 225 | 8.17 | 4.16 | 1.89 | γCH_2_  γCH_3_  δCH_3_ | 1.54, 1.23  0.92  0.88 |
| Glu 226 | 8.60 | 4.32 | 2.04 | γCH_2_ | 2.29 |
| Met 227 | 8.49 | 4.41 | 2.14 | γCH_2_  εCH_3_ | 2.66, 2.60  unassigned |
| Lys 228 | 8.39 | 4.04 | 1.86 | γCH_2_  δCH_2_  εCH_2_  εNH_3_^+^ | 1.47  1.72  2.97  unassigned |
| Lys 229 | 7.94 | 4.18 |  | γCH_2_  δCH_2_  εCH_2_  εNH_3_^+^ | 1.29  1.68  2.92  unassigned |
| His 230 | 7.99 | 4.50 | 3.00,  2.77 | 2H  4H | unassigned  7.10 |
| Tyr 231 | 7.81 | 4.33 | 3.21,  3.14 | 2,6H  3,5H | 7.16,  unassigned |
| Gln 232 | 8.27 | 4.07 | 2.20 | γCH_2_  δNH_2_ | 2.49  8.09, 7.04 |
| Val 233 | 7.74 | 3.86 | 2.19 | γCH_3_ | 1.05, 0.92 |
| Phe 234 | 7.30 | 4.47 | 3.21,  3.17 | 2,6H  3,5H  4H | unassigned  unassigned  unassigned |
| Glu 235 | 8.34 | 4.25 | 2.05 | γCH_2_ | 2.29 |
| Ala 236 | 7.97 | 4.12 | 1.48 | - |  |
| Lys 237 | 8.11 | 4.11 | 1.75 | γCH_2_  δCH_2_  εCH_2_  εNH_3_^+^ | 1.31  1.57  2.87  7.21 |
| Phe 238 | 7.84 | 4.45 | 3.20 | 2,6H  3,5H  4H | unassigned  unassigned  unassigned |
| Leu 239 | 7.90 | 4.09 | 1.80 | γH  δCH_3_ | 1.62  0.93 |
| Ser 240 | 7.87 | 4.29 | 3.98 | - |  |
| Lys 241 | 7.72 | 4.33 | 1.94,  1.82 | γCH_2_  δCH_2_  εCH_2_  εNH_3_^+^ | 1.45  1.66  2.97  7.17 |
| Ille 242 | 7.47 | 4.31 | 1.91 | γCH_2_  γCH_3_  δCH_3_ | 1.57, 1.15  0.92  0.84 |
| Pro 243 | - | 3.98 | 2.30,  2.00 | γCH_2_  δCH_2_ | 2.09  3.61 |
| His 244 | 7.90 | 4.47 | 3.20 | 2H  4H | unassigned  7.10 |
